# Reactive spinal glia convert 2-AG to prostaglandins to drive aberrant astroglial calcium signaling

**DOI:** 10.3389/fncel.2024.1382465

**Published:** 2024-05-09

**Authors:** Klaudia Dócs, Anita Balázs, Ildikó Papp, Peter Szücs, Zoltán Hegyi

**Affiliations:** ^1^Department of Anatomy, Histology and Embryology, Faculty of Medicine, University of Debrecen, Debrecen, Hungary; ^2^Department of Theoretical and Integrative Health Sciences, Institute of Health Sciences, Faculty of Health Sciences, University of Debrecen, Debrecen, Hungary; ^3^HUN-REN-DE Neuroscience Research Group, University of Debrecen, Debrecen, Hungary

**Keywords:** CB1, 2-AG, COX-2, cannabinoid, prostaglandin, calcium signaling, astrocyte, reactive astrocyte

## Abstract

The endogenous cannabinoid 2-arachidonoylglycerol (2-AG) influences neurotransmission in the central nervous system mainly by activating type 1 cannabinoid receptor (CB1). Following its release, 2-AG is broken down by hydrolases to yield arachidonic acid, which may subsequently be metabolized by cyclooxygenase-2 (COX-2). COX-2 converts arachidonic acid and also 2-AG into prostanoids, well-known inflammatory and pro-nociceptive mediators. Here, using immunohistochemical and biochemical methods and pharmacological manipulations, we found that reactive spinal astrocytes and microglia increase the expression of COX-2 and the production of prostaglandin E2 when exposed to 2-AG. Both 2-AG and PGE2 evoke calcium transients in spinal astrocytes, but PGE2 showed 30% more efficacy and 55 times more potency than 2-AG. Unstimulated spinal dorsal horn astrocytes responded to 2-AG with calcium transients mainly through the activation of CB1. 2-AG induced exaggerated calcium transients in reactive astrocytes, but this increase in the frequency and area under the curve of calcium signals was only partially dependent on CB1. Instead, aberrant calcium transients were almost completely abolished by COX-2 inhibition. Our results suggest that both reactive spinal astrocytes and microglia perform an endocannabinoid-prostanoid switch to produce PGE2 at the expense of 2-AG. PGE2 in turn is responsible for the induction of aberrant astroglial calcium signals which, together with PGE2 production may play role in the development and maintenance of spinal neuroinflammation-associated disturbances such as central sensitization.

## Introduction

Physiological pain plays an important role in protecting the individual from actual or potential tissue injuries. However, various pathological conditions, such as arthritis, diabetes, chemotherapy or inflammation frequently lead to chronic pain, characterized by allodynia, hyperalgesia and persistent pain sensation (Basbaum et al., 2009; Kuner, 2010). Since nociceptive information processing and transmission can exclusively be implemented by neural functions without any glial contribution, chronic pain is usually considered as an expression of maladaptive neural plasticity (Costigan et al., 2009; Luo et al., 2014). Indeed, in the spinal dorsal horn, which represents the primary relay station of the pain pathway, plasticity at synapses between nociceptors and spinal neurons in chronic pain plays an apparent role in the pathogenesis of chronic pain (Todd, 2010; Zeilhofer et al., 2012; Braz et al., 2014).

Importantly, however, central glial cells including astrocytes and microglia have been shown to influence a wide range of neural functions including nociception through neuronal-glial and glial-glial interactions (Watkins et al., 2001; Volterra and Steinhäuser, 2004; Scholz and Woolf, 2007; Verkhratsky, 2010; Graeber and Christie, 2012). Astroglial cells express neurotransmitter receptors and exhibit Ca^2+^ transients in response to glutamate, GABA, ATP and also endocannabinoids released by neurons (Perea et al., 2009; Parpura and Verkhratsky, 2012; Zorec et al., 2012; Bazargani and Attwell, 2016). Therefore, astrocytes encode and decode neuronal information by exhibiting Ca^2+^ transients of various frequency and amplitude depending on the neighboring synaptic activities (Volterra et al., 2014). Moreover, as a consequence of the intracellular Ca^2+^ elevations, astrocytes release various chemical substances termed as gliotransmitters to modulate the function of closely located neurons and glial cells (Araque et al., 2001; Halassa and Haydon, 2010; Parpura and Zorec, 2010; Parpura and Verkhratsky, 2012; Harada et al., 2015). Microglia also express receptors for various neurotransmitters which can maintain their quiescent state or promote a phenotypic switch (Pocock and Kettenmann, 2007; Liu et al., 2016). Microglia, on the other hand, can also release bioactive molecules such as cytokines and chemokines, thereby influencing synaptic transmission (Marinelli et al., 2019). Moreover, there is an extensive cross-talk between astrocytes and microglia due to their broad receptor and transmitter repertoire, underlying the complexity of communication and modulation of neuronal-glial assemblies (Matejuk and Ransohoff, 2020). Interestingly, some of the glia-related substances, such as endocannabinoids are among the most powerful antinociceptive transmitters (Hohmann, 2002; Rice et al., 2002; Hohmann and Suplita, 2006), whereas prostaglandins, for instance, are known to induce pronociceptive effects (Chapman and Dickenson, 1992; Bär et al., 2004; Heinricher et al., 2004; Kuner, 2010). Therefore, depending on their functional properties and actual phenotype, astrocytes and microglia can promote either a decrease or an increase of pain transmission.

Non-reactive spinal astrocytes and microglia express a complete molecular repertoire for the metabolism of, and responding to, endogenous cannabinoids (Hegyi et al., 2009, 2012; Dócs et al., 2015). In astrocytes, activation of CB1 receptors induces an elevation of intracellular Ca^2+^, which in turn activates diacylglycerol lipase alpha (DGL-α), biosynthetic enzyme of 2-AG, in the same cell, resulting in cannabinoid-induced 2-AG mobilization (Navarrete and Araque, 2008; Hegyi et al., 2018). The released 2-AG is very likely to exert an antinociceptive effect at spinal levels (Hama and Urban, 2004), partly through autocrine and paracrine signaling to glial cells in the diffusion range, and partly through the induction of a CB1-mediated decrease of synaptic release probability (Morisset and Urban, 2001). Endocannabinoids also activate CB2 receptors expressed by microglia, promoting a switch from M1 to M2 phenotype. In fact, microglial CB2 drives a release of anti-inflammatory and neuroprotective mediators such as IL-10 and 2-AG (Luongo et al., 2010; Tanaka et al., 2020).

Various stimuli, however, may induce profound alterations in the morphology and gene expression pattern of central glia, resulting in the formation of reactive astrocytes and reactive microglia (Pekny and Pekna, 2014; Liddelow and Barres, 2017; Wolf et al., 2017; Gao et al., 2023).

Considering its central role in pain processing, overexpression of cyclooxygenase-2 (COX-2) is one of the most intriguing alterations in neuroinflammatory astrocytes and microglia (Molina-Holgado et al., 2000; Brambilla et al., 2002; Minghetti, 2004; Font-Nieves et al., 2012). COX-2 metabolizes arachidonic acid to produce various prostaglandins including prostaglandin E2 (PGE2), which is among the best-known mediators that induce and maintain inflammatory pain (Woolf and Salter, 2000; Heinricher et al., 2004; Liang et al., 2007). Arachidonic acid, the primary substrate for COX-2 is provided mostly by phospholipases such as PLA2 and PLC, which liberate arachidonic acid from membrane lipids. Additional biochemical routes, however, also yield arachidonic acid, serving as alternative sources of prostanoid biosynthesis. One of these mechanisms is the canonical degrading pathway of the most important endocannabinoids, anandamide and 2-AG, which, during physiological conditions, are mostly hydrolyzed by FAAH (fatty acid amid hydrolase) and MGL (monoglycerol lipase) to yield arachidonic acid (Muccioli, 2010). Therefore, COX-2 represents an important link between endocannabinoid and prostanoid signaling. Moreover, COX-2 has a broad substrate specificity, and directly accepts anandamide and 2-AG, which are converted into prostaglandin-ethanolamides and prostaglandin-glycerol esters, respectively (Fowler, 2007; Rouzer and Marnett, 2011). Direct oxidation of endocannabinoids by COX-2 indicates that, with or without a prior hydrolysis, 2-AG will drive prostaglandin synthesis.

Astrocytes respond to both 2-AG and PGE2 with Ca^2+^ transients. Therefore, effects of endocannabinoid and prostaglandin signaling on astroglial calcium elevations can be additive. It is not known, however, how astrocytes and microglia with different phenotypes participate in the COX-2 mediated oxidative degradation of 2-AG, and how COX-2-derived metabolites influence the effects of 2-AG. Thus, here we investigated the extent of conversion of 2-AG to PGE2 in central glia, and the impact of 2-AG oxygenation on the evoked biological responses in non-reactive astrocytes, as well as the alterations of these mechanisms in inflammatory conditions.

## Results

### Expression of DGL-α, CB1 and COX-2 in the spinal dorsal horn are altered in response to peripheral inflammation

The spinal dorsal horn (SDH) represents the primary relay station of pain pathway (Todd, 2010). Accordingly, maladaptive structural and functional plasticities in the superficial laminae of the spinal cord play a substantial role in the development and maintenance of persistent pain (Kuner, 2010; Luo et al., 2014). We have previously shown that DGL-α and CB1, the enzyme producing 2-AG and the receptor mediating its effect in the central nervous system, are expressed by neurons and glial cells in the SDH (Hegyi et al., 2009, 2012). COX-2 has also been demonstrated on spinal neurons, astrocytes and microglia (Ghilardi et al., 2004; Wang et al., 2011). As a first step of our investigation, we asked if peripheral inflammation alters the expression level and pattern of these molecules in SDH of mice.

**Figure 1:**
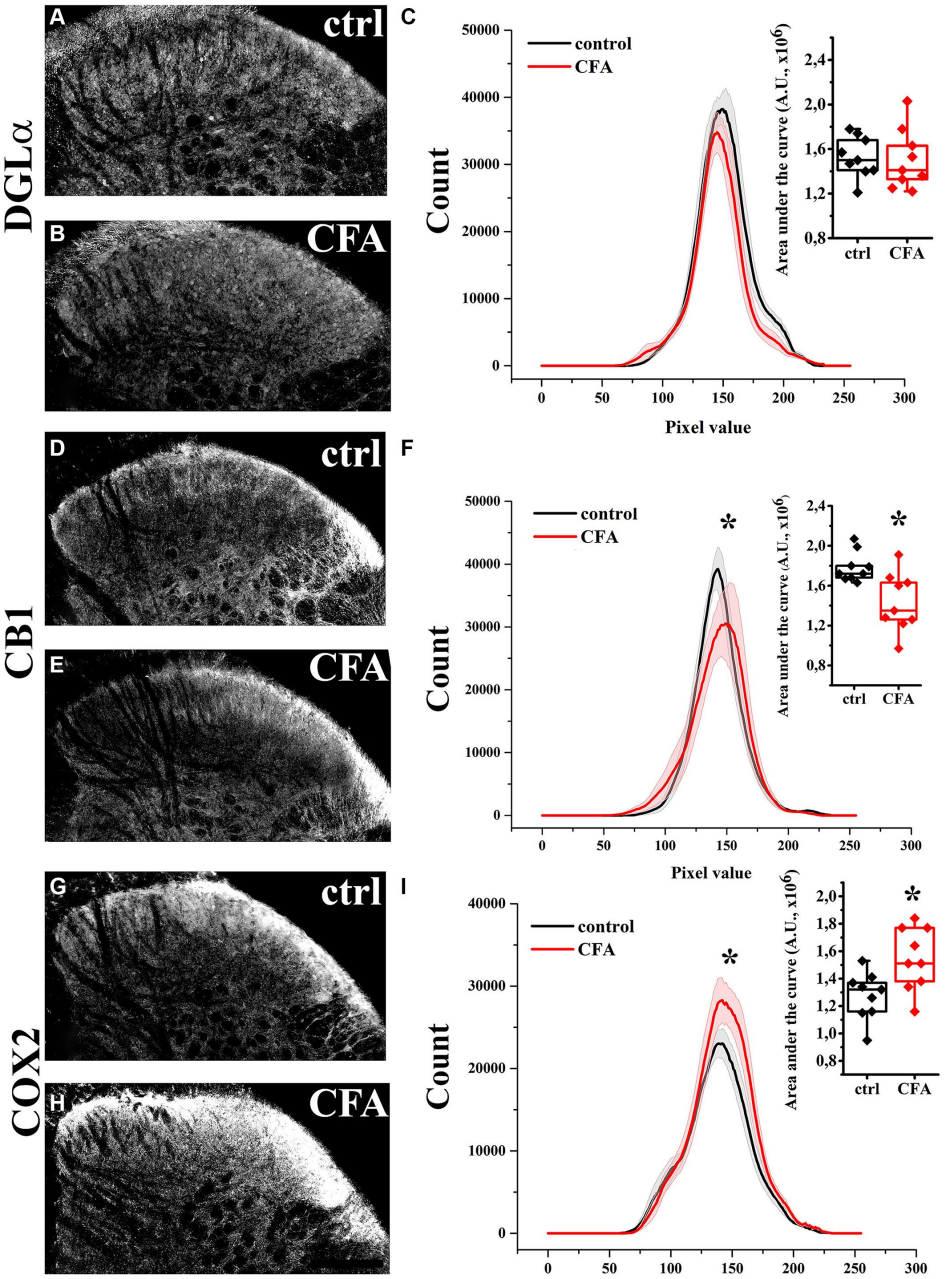
Immunohistochemical localization and its histogram profile of DGL-α, CB1 and COX-2 in control and CFA-treated animals. Following a CFA treatment, DGL-α immunostaining showed an unaltered intensity in the SDH of mice **(A–C)**. CFA decreased CB1 **(D–F)** but increased COX-2 **(G–I)** expression in the SDH **(A–F)**. *n* = 9 spinal dorsal horns. Box plot properties: center: median; edges: 25th–75th percentiles; whiskers: extrema; outliers plotted individually. Statistical comparison: Kolmogorov–Smirnov test. Scale bar: 100 μm.

In agreement with our previous results, DGL-α showed a strong immunostaining in the SDH with a heavily stained band in lamina I and much weaker staining in all deeper laminae (Hegyi et al., 2012). Following a treatment with Complete Freund’s Adjuvant (CFA), DGL-α showed a more dispersed staining, but its immunoreactivity in SDH altogether did not change significantly (*p* = 0.658; Figures 1A–C; Table 1).

**Table 1:** AUC values of histogram profiles of SDHs of naïve and CFA-treated mice immunostained for DGL-α, CB1 and COX-2.

	AUC (AU, x10^6^)		
	naïve	CFA	
DGL-α	1.53 ± 0.06	1.5 ± 0.09	*p = 0.658*
CB1	1.79 ± 0.05	1.43 ± 0.1	*p = 0.007*
COX-2	1.21 ± 0.06	1.55 ± 0.8	*p = 0.003*

Similar to our previous data, immunostaining for CB1 showed a twin-band in the SDH confined to lamina I and the inner segment of lamina II (Hegyi et al., 2009). On day 4 following CFA treatment, the characteristics of CB1 immunostaining did not change profoundly in SDH, but its intensity decreased by 21% (*p* = 0.007; Figures 1D–F; Table 1).

The SDH of naïve mice showed a relatively weak and diffuse COX-2 immunoreactivity, which appeared to be moderately heavier in the superficial laminae (Figure 1G). On day 4 of CFA-induced peripheral inflammation, the expression of COX-2 in SDH increased by 28% (*p* = 0.003; Figures 1H,I; Table 1). Some of the immunostained elements appeared to be cell bodies, but the majority of immunostaing showed a fine punctate pattern. These data suggest that, under inflammatory conditions, quantities of released 2-AG may not be altered profoundly. However, the probability that 2-AG is converted to prostaglandins instead of activating CB1 receptors is significantly increased.

### Astrocytes and microglia in the spinal dorsal horn increase the expression of COX-2 following peripheral inflammation

Spinal astrocytes are extensively involved in cannabinoid signaling by releasing and responding to the endocannabinoid 2-AG (Hegyi et al., 2018; Duffy et al., 2021). Microglia has also been shown to express COX-2 (de Oliveira et al., 2008; Gong et al., 2008; Schlachetzki et al., 2010). The expression of COX-2 in astrocytes, however, seems much less straightforward in the literature. Astrocytes were found to be lacking COX-2 in previous publications (Anrather et al., 2011; Avraham et al., 2015), whereas others confirmed the expression of COX-2 by astrocytes (Wu et al., 2011; Kaizaki et al., 2013; Hsieh et al., 2018). To determine the possible role of spinal glia in the oxidative metabolism of 2-AG, we next investigated the expression of COX-2 in SDH astrocytes and microglia in naïve and CFA-treated mice.

**Figure 2:**
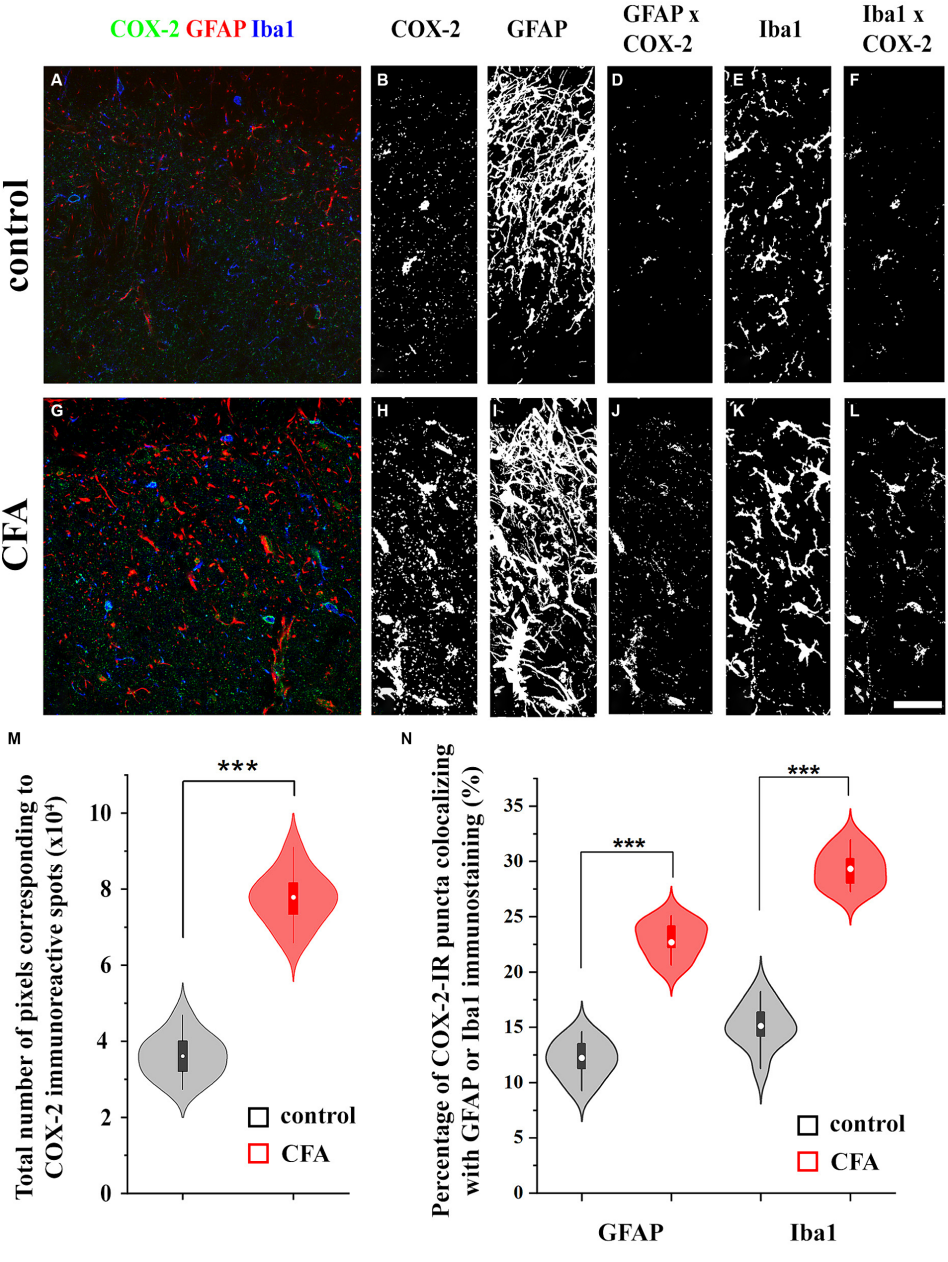
Astrocytes and microglial cells in the spinal dorsal horn increase the expression of COX-2 following a CFA treatment. Micrographs of single 1-μm-thick confocal optical sections showing the co-localization between astrocytes (GFAP-positive profiles, red) or microglial cells (Iba1-positive profiles, blue) and COX-2 immunostained spots (green) in the SDH of naive **(A)** and CFA-treated mice **(G)**. Z-projection of 15 1-μm-thick confocal optical sections to show the density of COX-2 immunoreactive puncta, GFAP and Iba1-immunostained profiles in naïve (**B**,**C**,**E**, respectively) and CFA-treated animals (**H**,**I**,**K**, respectively). Note that the number of pixels corresponding to COX-2 immunostaining **(M)** and their co-localization with GFAP or Iba1 positive profiles are significantly increased in the SDH of CFA-treated mice (**D** vs. **J**,**F** vs. **L**,**N**). *n* = 9 spinal dorsal horns. Box plot properties: center: median; edges: 25th–75th percentiles; whiskers: extrema; outliers plotted individually. Statistical comparison: two-tailed non-parametric Mann–Whitney U test. Scale bar: 5 μm.

Similar to single immunohistochemistry, double immunofluorescence revealed 3.32 ± 0.91 × 10^4^ pixels corresponding to COX-2 immunostaining in the SDH of naïve mice (Figures 2A,B,M; Supplementary Figures 1A,B,M), of which 12.41 ± 2.79% and 15.64 ± 3.02% co-localized with astrocytes (GFAP-positive profiles; Figures 2C,D,N; Supplementary Figures 1C,D,N) and microglia (Iba1-immunostained profiles; Figures 2E,F,N; Supplementary Figures 1E,F,N), respectively. Following a CFA treatment, the number of pixels for COX-2 staining increased by 240%, to 7.98 ± 1.67 × 10^4^ pixels (Figures 2G,H,M; Supplementary Figures 1G,H,M), of which 22.4 ± 3.77% and 28.91 ± 4.12% co-localized with GFAP (Figures 2I,J,N; Supplementary Figures 1I,J,N) and Iba1 (Figures 2K,L,N; Supplementary Figures 1K,L,N) immunostained elements, respectively. Based on these data, CFA treatment induced a 4-fold increase in COX-2 expression in spinal glia (astrocytes and microglia together, from ~0.92 × 10^4^ to ~4.23 × 10^4^ pixels) suggesting that, in inflammatory conditions, these cells may effectively participate in the conversion of 2-AG to prostaglandines.

### The expression of COX-2 is increased in spinal astrocyte-microglia co-culture following an LPS treatment

Astrocyte and microglia profoundly change their expression phenotype and quickly undergo an inflammatory transition in response to an LPS treatment (Cunningham et al., 2019; Wendimu and Hooks, 2022; Lawrence et al., 2023). Therefore, as the subsequent experiments were carried out on primary spinal astrocyte-microglia co-cultures, neuroinflammatory response of glial cells was induced by an LPS stimulus.

**Figure 3:**
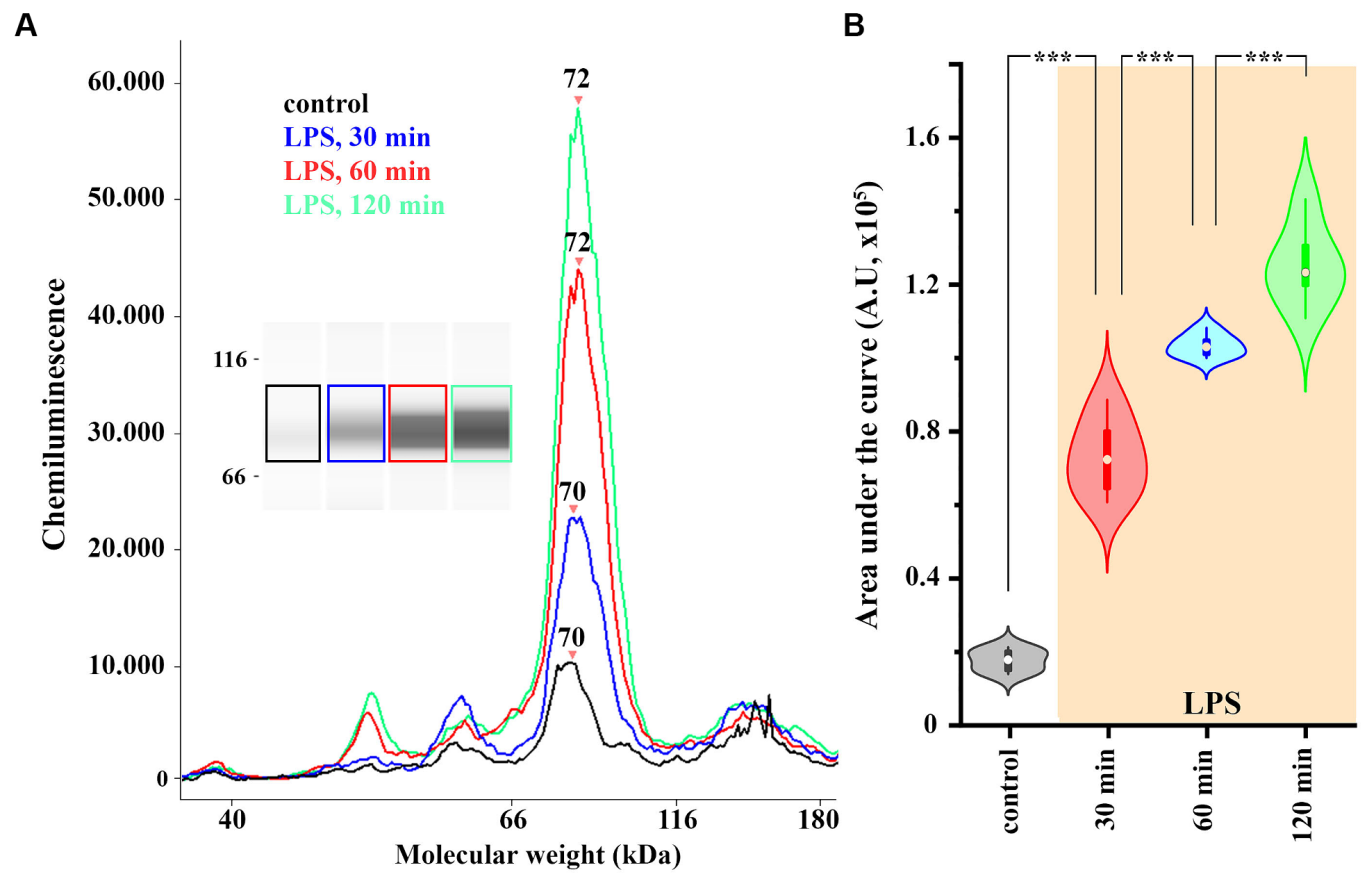
The expression of COX-2 is increased in spinal astrocyte-microglia co-culture **(A,B)**. A representative image of a capillary Western blot (WES) analysis of COX-2 expression in control and LPS-treated astrocyte-microglia co-cultures **(A,B)**. *n* = 9 cultures. Box plot properties: center: median; edges: 25th–75th percentiles; whiskers: extrema; outliers plotted individually. Statistical comparison: two-way Anova with Bonferroni correction.

To detect global, LPS-induced changes in the level of COX-2 expression in spinal glia, we performed a capillary electrophoresis immunoassay (WES) on control and LPS-treated astrocyte-microglia co-cultures. To demonstrate the time-course of the induction of COX-2 expression, LPS was applied for 30, 60 and 120 min, which increased COX-2 expression from 1.69 ± 0.12 AU to 7.27 ± 0.42 (*p* < 0.005), 10.3 ± 0.13 (*p* = 0.005) and 12.49 ± 0.46 AU (*p* = 0.005), respectively (Figures 3A,B). In the subsequent experiments, an LPS stimulus for 120 min was applied to induce the expression of COX-2.

### Both reactive spinal astrocyte and microglia increase the expression of COX-2 in primary culture

**Figure 4:**
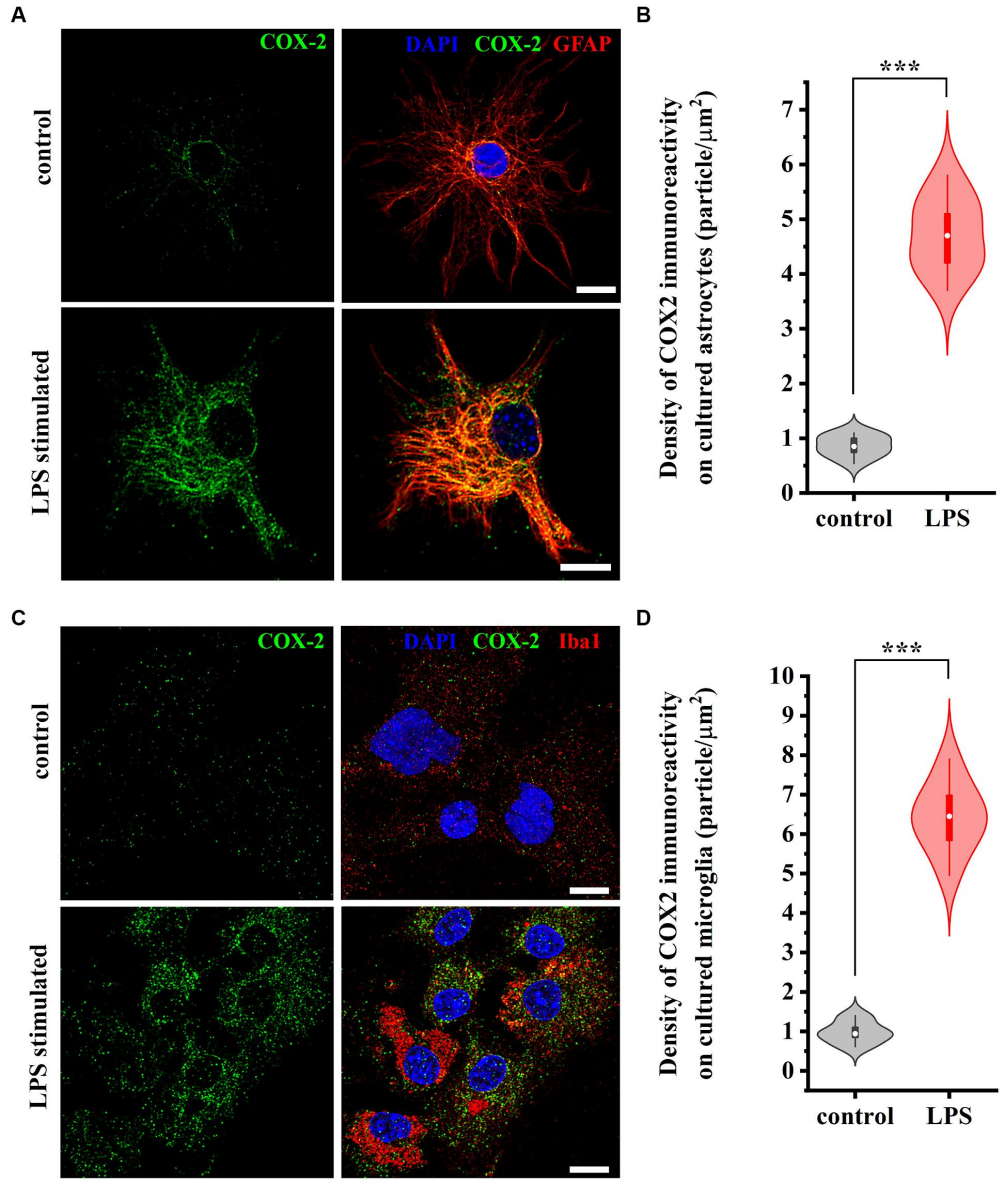
Micrographs of immunostained control and LPS-treated spinal astrocytes-microglia co-cultures illustrating that reactive spinal astrocytes and microglia increase the expression of COX-2 following an LPS stimulus. Both astrocytes (GFAP, red, **A**) and microglia (Iba1, red, **C**) increase the expression of COX-2 (green, **A,C**) following a treatment with LPS **(B,D)**. *n* = 18 cells. Box plot properties: center: median; edges: 25th–75th percentiles; whiskers: extrema; outliers plotted individually. Statistical comparison: two-tailed non-parametric Mann–Whitney U test. Scale bar: 5 μm.

To verify that both astrocytes and microglia participate in the increased expression of COX-2 seen in WES analysis, we immunostained control and LPS-treated cell cultures for GFAP and Iba1, as well as for COX-2. In control conditions, 0.88 ± 0.12 and 1.12 ± 0.27 COX-2 / μm^2^ immunoreactive puncta were recovered on astrocytes (Figures 4A,B) and microglia (Figures 4C,D), respectively. LPS treatment for 2 h increased the number of COX-2 (immunreactive puncta to 4.88 ± 0.96 / μm^2^, *p* < 0.001; Figures 4A,B) in astrocytes and (to 6.35 ± 1.04 / μm^2^, *p* < 0.001; Figures 4C,D) in microglia.

### 2-AG drives PGE2 production in spinal glia

Astrocytes and microglia express DGL-α (Hegyi et al., 2012) and release 2-AG (Carrier et al., 2004; Hegyi et al., 2018). Our data showed that spinal astrocytes and microglia express COX-2 and, based on earlier data, they both can release PGE2 (Zhang et al., 2009; Clasadonte et al., 2011; Xia and Zhu, 2011). Considering that oxidative metabolism of 2-AG is driven at least partly by COX-2 resulting in the synthesis of PGE2 (Alhouayek and Muccioli, 2014), next, using a metabolite ELISA essay, we tested if control and LPS-treated spinal glia convert 2-AG to PGE2.

**Figure 5:**
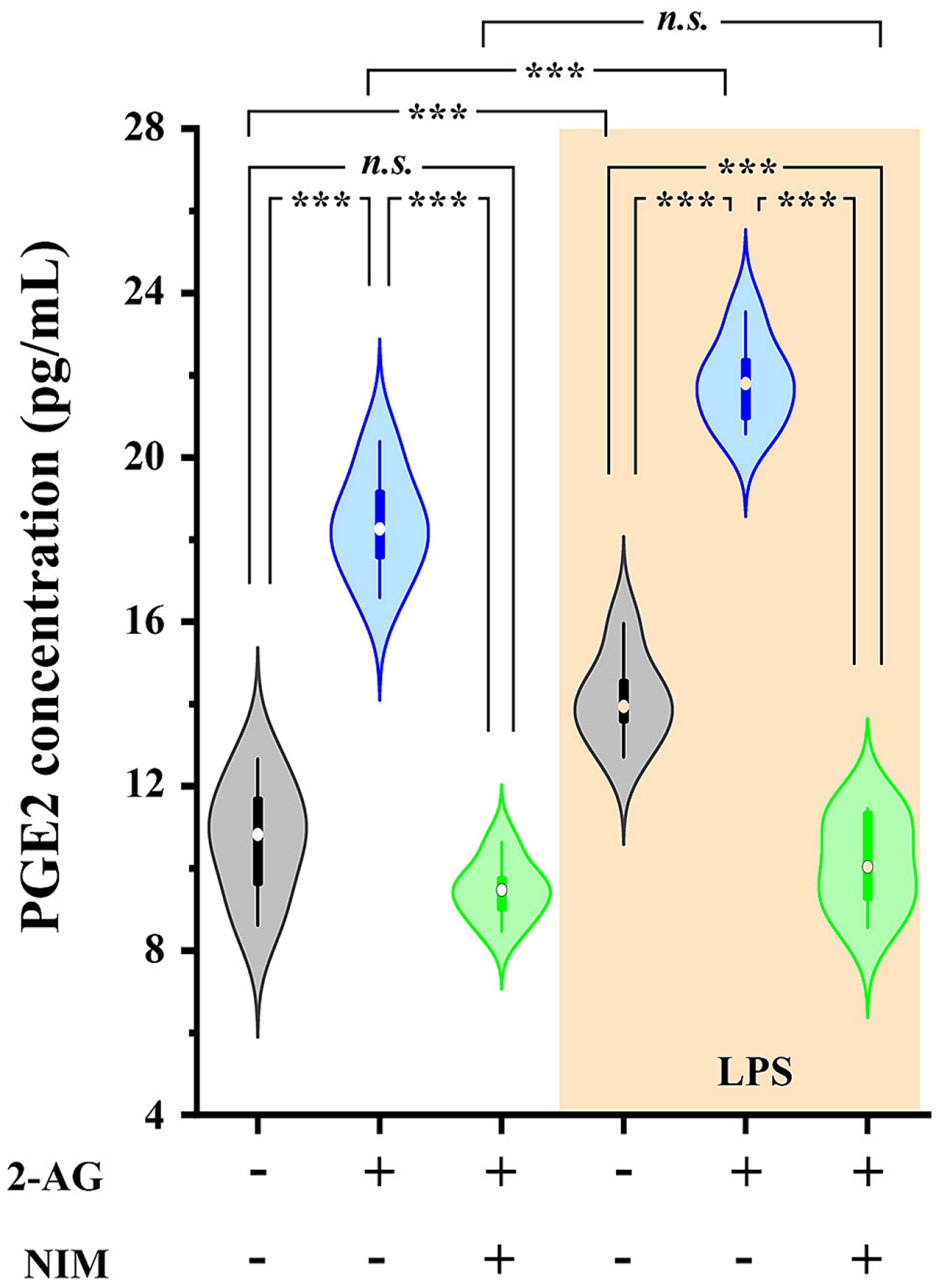
PGE2 production is increased in spinal astrocyte-microglia co-cultures in the presence of 2-AG in both control and LPS-treated cultures. Application of 2-AG does not alter PGE2 production in the presence of the COX-2 inhibitor nimesulide. *n* = 9 cultures. Box plot properties: center: median; edges: 25th–75th percentiles; whiskers: extrema; outliers plotted individually. Statistical comparison: two-way Anova with Bonferroni correction. n.s: not significant. ***p < 0.001.

Untreated astrocyte culture contained 10.71 ± 0.72 pg./mL PGE2 which increased to 14.11 ± 0.33 pg./mL (*p* < 0.001) following a treatment with LPS. We next administered 200 pmol 2-AG to the media, which in 5 min increased PGE2 concentration to 18.37 ± 0.66 pmol/mL in control (*p* < 0.001, vs. untreated basal values) and 21.84 ± 0.32 pmol/mL in LPS-treated cultures (*p* < 0.001, vs. LPS basal values). Pretreating the cultures with the COX-2 inhibitor nimesulide at 100 μM prior to the application of 2-AG decreased PGE2 levels to 9.47 ± 0.24 (*p* < 0.001, vs. 2-AG-treated cells) and 10.12 ± 0.44 (*p* < 0.001, vs. 2-AG-treated LPS-stimulated cells; Figure 5). These results confirm that the increased expression of COX-2 in reactive spinal glia allows an increased conversion of 2-AG to prostaglandines.

### Both 2-AG and PGE2 induce Ca^2+^ transients in spinal astrocytes

Astrocytes in SDH express CB1 (Hegyi et al., 2009) which mediate the effects of 2-AG and induces an increase in the intracellular Ca^2+^ concentration (Hegyi et al., 2018). Astrocytes also possess prostaglandin EP3 receptor (Takemiya and Yamagata, 2013) and respond to PGE2 with Ca^2+^ transients (Takemiya et al., 2011). Because the efficacy and potency of 2-AG and PGE2 can be different at inducing Ca^2+^ transients, conversion of 2-AG into PGE2 may increase astroglial calcium signaling following 2-AG mobilization. Thus, we next recorded the changes in intracellular Ca^2+^ concentration of astrocytes in untreated spinal astrocyte-microglia co-cultures following the application of 2-AG and PGE2 from 10 pM to 1 mM and illustrated the AUC of calcium signals as the function of the concentration of ligands to obtain standard concentration-response curves. To check the viability of cells, 180 μM ATP was applied at the end of the measurement which has been shown to induce a robust Ca^2+^ transient (Hegyi et al., 2018).

**Figure 6:**
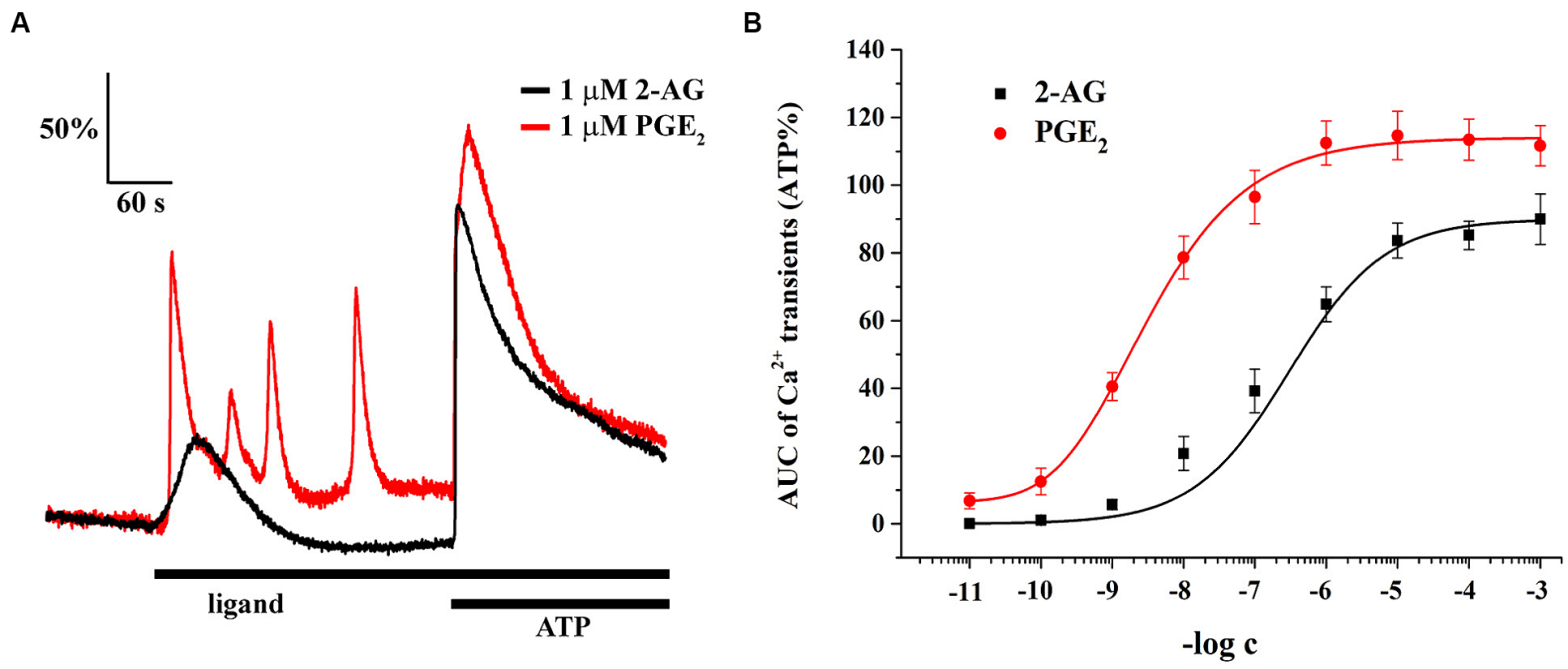
Both 2-AG and PGE2 induce calcium transients in spinal cultured astrocytes. Equimolar concentration of PGE2 is more potent and efficacious than 2-AG at inducing calcium transients **(A,B)**. *n* = 18 cells.

Astrocytes showed Ca^2+^ signals in response to both ligands, with a notable differences at equimolar concentrations regarding the pattern and AUC of the transients, normalized to the AUC of ATP-induced transients (Figure 6A; Table 2). The lowest concentration of 2-AG which induced Ca^2+^ transients was found to be in the nanomolar range, whereas the maximal response evoked by 2-AG was 89.98 ± 7.42% of that of ATP, with an EC50 value of 0.16 μM (Figure 6B). In agreement with our current calculation, EC50 of 2-AG previously was found to be 0.6 μM on CB1 transfected COS7 cells (Dócs et al., 2017). Increasing concentrations of 2-AG and PGE2 elevated the frequency of induced Ca^2+^ transients from 0 to 1.88 ± 0.23 / minute and from 2.01 ± 0.19 to 3.1 ± 0.49 / minute, respectively (Table 3).

**Table 2:** AUC values of Ca^2+^ transients induced by 2-AG and PGE2.

Concentration	AUC (ATP%)		*p*
	2-AG	PGE2	
1 mM	89.98 ± 7.42	111.69 ± 5.97	<0.001
100 μM	85.19 ± 4.23	113.45 ± 6.11	<0.001
10 μM	83.67 ± 5.12	114.67 ± 7.12	<0.001
1 μM	64.84 ± 5.11	112.46 ± 6.5	<0.001
100 nM	39.24 ± 6.45	96.54 ± 7.88	<0.001
10 nM	20.81 ± 4.99	78.65 ± 6.33	<0.001
1 nM	5.66 ± 1.43	40.51 ± 4.09	<0.001
100 pM	1.1 ± 0.4	12.47 ± 3.91	0.004
10 pM	0	6.79 ± 2.31	0.004

**Table 3:** Frequency of Ca^2+^ transients induced by 2-AG and PGE2.

Concentration	Frequency (1/min)		*p*
	2-AG	PGE2	
1 mM	1.88 ± 0.23	3.1 ± 0.49	<0.001
100 μM	1.79 ± 0.27	3.16 ± 0.56	<0.001
10 μM	1.74 ± 0.24	2.99 ± 0.47	<0.001
1 μM	1.66 ± 0.19	2.87 ± 0.39	<0.001
100 nM	1.27 ± 0.11	2.81 ± 0.44	<0.001
10 nM	1.01 ± 0.1	2.79 ± 0.34	<0.001
1 nM	0.87 ± 0.1	2.43 ± 0.27	<0.001
100 pM	0.31 ± 0.07	2.19 ± 0.23	<0.001
10 pM	0	2.01 ± 0.19	<0.001

PGE2 evoked Ca^2+^ transients at 10 pM, and its maximal effect induced at 1 uM was 114.67 ± 7.12% of ATP response. EC50 of PGE2 was calculated to be 2.98 nM, 55 times lower than that of 2-AG (Figure 6B). These data indicate that an increased expression of COX-2 in glial cells, through the conversion of 2-AG to PGE2, may indirectly lead to an exaggerated calcium signaling in spinal astrocytes.

### 2-AG-induced exaggerated Ca^2+^ signals in reactive spinal astrocytes depend on 2-AG—PGE2 conversion

Aberrant astroglial Ca^2+^ signals have been linked to certain neuropathological conditions (Dong et al., 2018; Shigetomi et al., 2019; Bancroft and Srinivasan, 2021), and they also play role in pain processing (Cho and Huh, 2020; Xu et al., 2021). Due to the capacity of spinal glia to convert 2-AG to PGE2, exposing these cells to 2-AG may lead to a PGE2-mediated alteration of astroglial Ca^2+^ signaling, an effect that may be exacerbated in neuroinflammatory astrocytes. Thus, we investigated spontaneous and 500 nM 2-AG-induced Ca^2+^ transients of astrocytes in control and LPS-treated astrocyte-microglia co-cultures.

**Figure 7:**
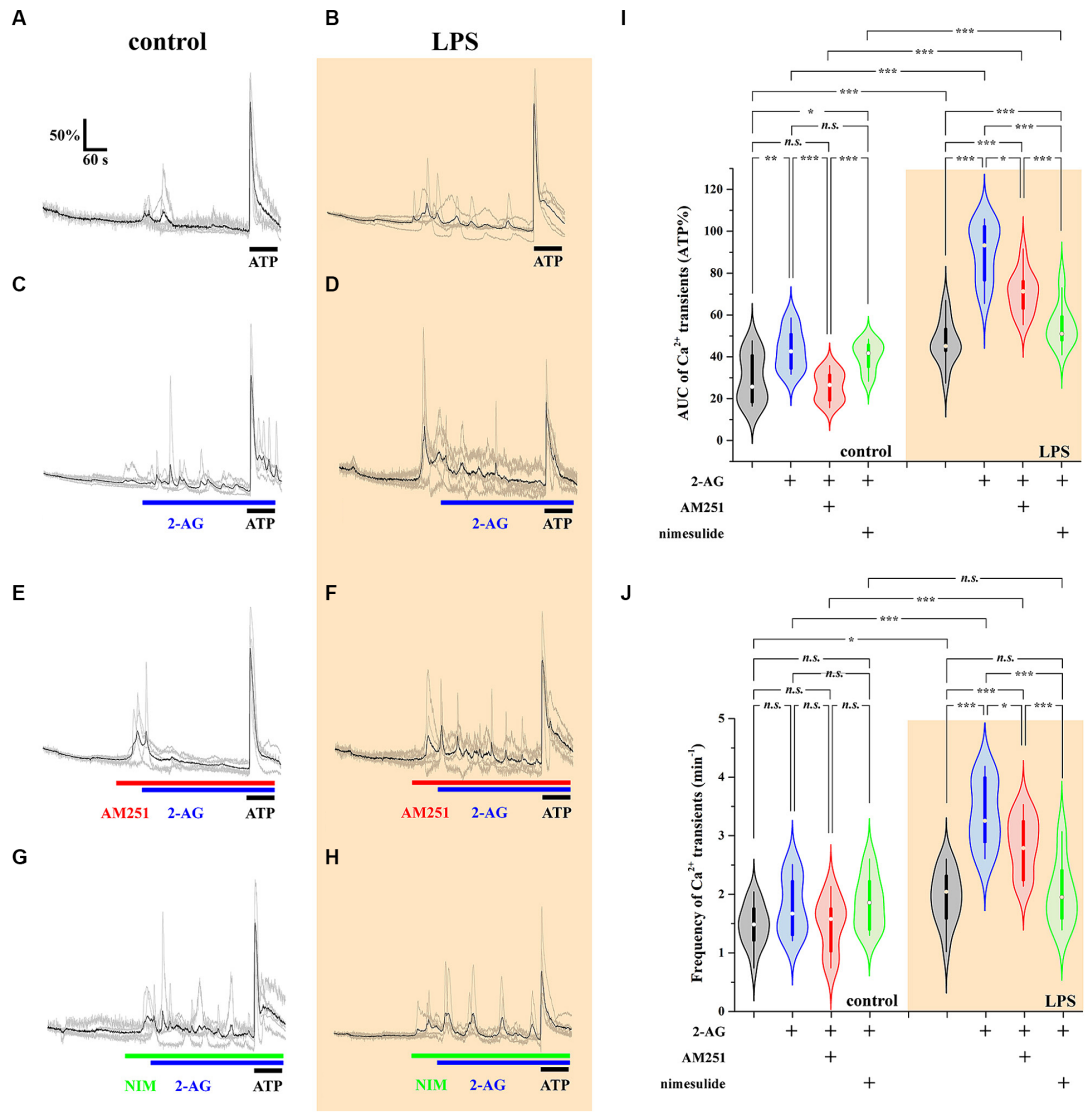
2-AG induced exaggerated calcium signals in reactive astrocytes are dependent on COX-2 activity **(A–J)**. Calcium transients following the application of 2-AG are fully prevented by the inhibition of CB1, but not affected by the inhibition of COX-2 in control astrocytes **(A,C,E,G,I,J)**. Inhibition of CB1 only partially, but inhibition of COX-2 almost fully prevents the induction of calcium signals in LPS-treated astrocytes **(B,D,F,H,I,J)**. *n* = 18 cells. Box plot properties: center: median; edges: 25th–75th percentiles; whiskers: extrema; outliers plotted individually. Statistical comparison: two-way Anova with Bonferroni correction. n.s, not significant. *p < 0.05; **p < 0.005; ***p < 0.001.

The AUC, but not the frequency, of Ca^2+^ transients in control astrocytes was increased by 60% following the application of 2-AG (Figures 7A,C,I,J; Tables 4, 5). In reactive astrocytes, both AUC and frequency of spontaneous Ca^2+^ activity were elevated by 71 and 40%, respectively, which were further increased after the application of 2-AG by 106 and 84% (Figures 7B,D,I,J; Tables 4, 5).

**Table 4:** AUC of Ca^2+^ transients in control and LPS-treated spinal astrocytes induced by 2-AG, with or without a pretreatment by AM251 or nimesulide.

	AUC (AU)		
	untreated	LPS	
Control	27.94 ± 3.47	48.20 ± 3.15	*p_(untreated vs. LPS)_ < 0.001*
2-AG	43.93 ± 2.89	99.60 ± 4.15	*p_(untreated vs. LPS)_ < 0.001*
	*p_(vs. control)_ = 0.005*	*p_(vs. control)_ < 0.001*	
AM251 + 2-AG	23.07 ± 2.10	76.20 ± 3.01	*p_(untreated vs. LPS)_ < 0.001*
	*p_(vs. control)_ = 0.43*	*p_(vs. control)_ < 0.001*	
	*p_(vs. 2-AG)_ < 0.001*	*p_(vs. 2-AG)_ = 0.016*	
nimesulide + 2-AG	40.47 ± 2.10	57.67 ± 3.09	*p_(untreated vs. LPS)_ < 0.001*
	*p_(vs. control)_ = 0.01*	*p_(vs. control)_ = 0.042*	
	*p_(vs. 2-AG)_ = 0.467*	*p_(vs 2-AG)_ < 0.001*	
	*p_(vs. AM251 + 2-AG)_ < 0.001*	*p_(vs. AM251 + 2-AG)_ < 0.001*	

**Table 5:** Frequency of Ca^2+^ transients in control and LPS-treated spinal astrocytes induced by 2-AG, with or without a pretreatment by AM251 or nimesulide.

	Frequency (1/min)		
	Untreated	LPS	
Control	1.28 ± 0.09	1.8 ± 0,13	*p_(untreated vs. LPS)_ = 0.004*
2-AG	1.64 ± 0.13	3.32 ± 0.16	*p_(untreated vs. LPS)_ < 0.001*
	*p_(vs. control)_ = 0.041*	*p_(vs. control)_ < 0.001*	
AM251 + 2-AG	1.29 ± 0.12	2.77 ± 0.14	*p_(untreated vs. LPS)_ < 0.001*
	*p_(vs. control)_ = 0.755*	*p_(vs. control)_ < 0.001*	
	*p_(vs 2-AG)_ = 0.145*	*p_(vs. 2-AG)_ = 0.037*	
nimesulide + 2-AG	1.7 ± 0.12	1.87 ± 0.15	*p_(untreated vs. LPS)_ = 0.0478*
	*p_(vs. control)_ = 0.049*	*p_(vs. control)_ = 0.917*	
	*p_(vs. 2-AG)_ = 0.617*	*p_(vs. 2-AG)_ < 0.001*	
	*p_(vs. AM251 + 2-AG)_ = 0.084*	*p_(vs. AM251 + 2-AG)_ < 0.001*	

Reactive spinal astrocytes responded to 2-AG with exaggerated Ca^2+^ signal, which may be related to an increased COX-2 activity and a consequent conversion of 2-AG to PGE2. To test this hypothesis, untreated and LPS-stimulated astrocyte cultures were pretreated with either the CB1 inverse agonist AM251 (5 μM) or the COX-2 inhibitor nimesulide (100 μM) prior to the application of 2-AG.

In resting astrocytes, prevention of CB1 mediated effects by AM251 resulted in a Ca^2+^ signaling similar to basal Ca^2+^ activities, that is, 2-AG this time had no effect on the intracellular Ca^2+^ concentration (Figures 7E,I,J; Table 4, 5). On the other hand, nimesulide pretreatment was unable to prevent 2-AG from inducing Ca^2+^ transients, and the evoked responses appeared to be practically identical to those without nimesulide (Figures 7G,I,J; Tables 4, 5).

**Figure 8:**
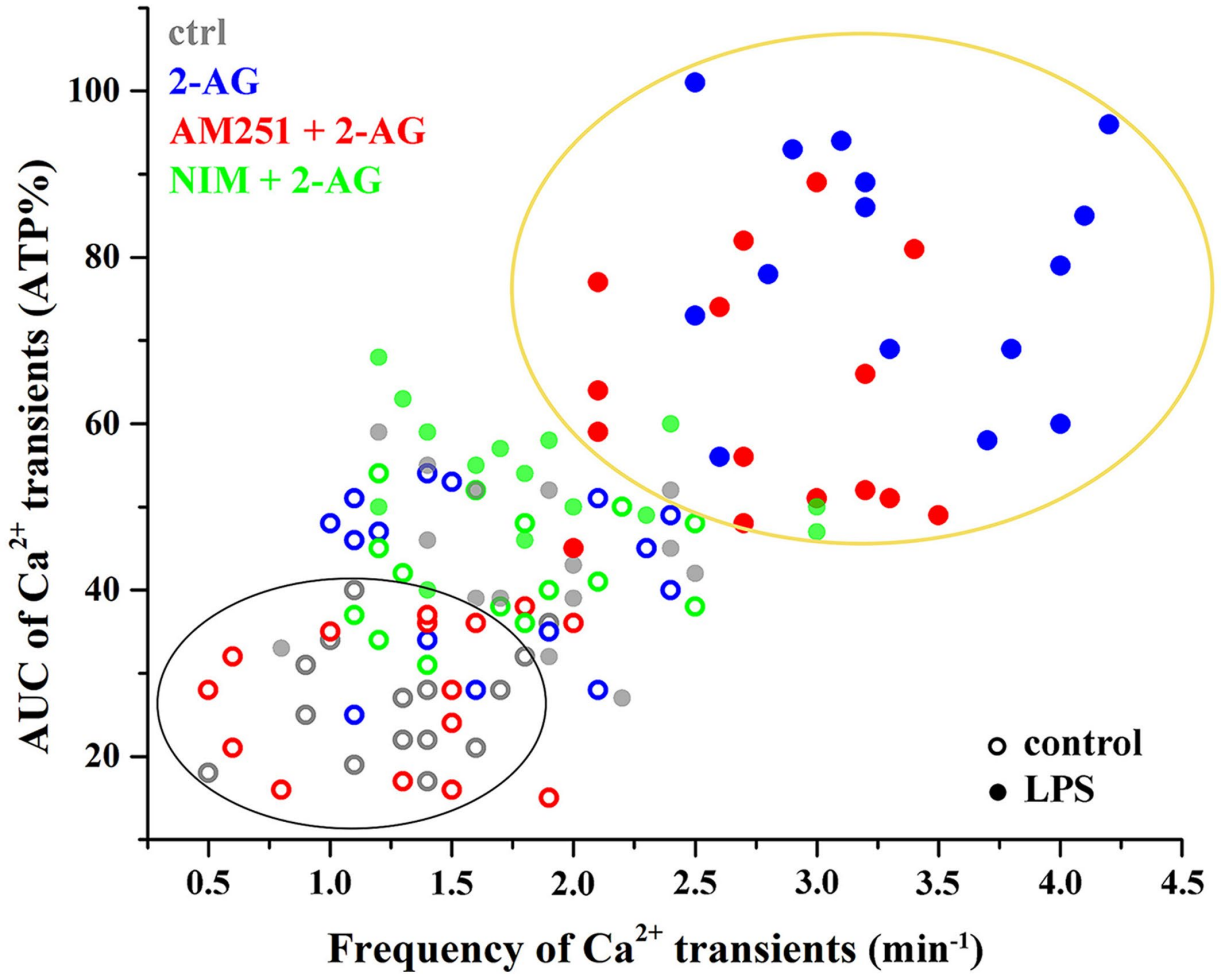
Clustering of calcium transients of control and LPS-treated astrocytes following the inhibition of CB1 or COX-2. Clusters and confidence ellipses were identified using K-means cluster analysis. The left bottom cluster indicates that the effects of 2-AG are abolished by the CB1 antagonist AM251 in resting cells. The top right cluster shows that, in LPS-treated cells, effects of 2-AG are abolished by the COX-2 inhibitor nimesulide, but not AM251.

Increased Ca^2+^ transients evoked by 2-AG in reactive astrocytes were only partially inhibited by AM251 (Figures 7F,I,J; Tables 4, 5). Nimesulide, however, almost completely abolished 2-AG-induced exaggerated Ca^2+^ transients (Figures 7H–J; Tables 4, 5). Plotting the AUC of Ca^2+^ signals as the function of their frequency revealed a clustering of responses of 2-AG-treated reactive astrocytes with or without AM251 pretreatment. Similarly, Ca^2+^ signals of resting astrocytes in response to 2-AG following AM251 pretreatment formed a cluster with spontaneous Ca^2+^ activities of unstimulated cells (Figure 8).

## Discussion

Central glia controls synaptic functions through releasing a plethora of bioactive substances from glutamate and ATP to interleukins and endocannabinoids (Volterra and Bezzi, 2002; Harada et al., 2015; Reemst et al., 2016; Dzyubenko and Hermann, 2023; Liu et al., 2023). The broad neurochemical repertoire of glial cells to influence neuronal and non-neuronal functions proved to be a double-edged sword, since reactive astrocytes and microglia profoundly change their expression profile and the composition of the released gliotransmitters (Pekny and Pekna, 2014; Bachiller et al., 2018; Bennett and Viaene, 2021; Escartin et al., 2021; Hasel et al., 2021; Woodburn et al., 2021). An appropriate set of gliotransmitters seems inevitable in maintaining neural functions, but substances released by reactive glia can be disadvantageous or even harmful for neural networks. In the past decades, accordingly, astrocytes and microglia have been linked not only to physiological processes of the central nervous system, but also to several neurodegenerative and neuroinflammatory diseases (Maragakis and Rothstein, 2006; Di Malta et al., 2012; Hickman et al., 2018; Muzio et al., 2021; Yang et al., 2021). Importantly, spinal astrocytes and microglia play a role in pain processing, and their role in persistent pain has also been demonstrated (Watkins et al., 2001; Gao and Ji, 2010; Ho et al., 2020; Xu et al., 2021; Gu et al., 2022).

Resting spinal astrocytes and microglia express a complete molecular toolbox of cannabinoid signaling to respond to, synthesize and degrade endogenous cannabinoid ligands including 2-AG (Hegyi et al., 2009, 2012; Dócs et al., 2015). Moreover, when exposed to 2-AG, spinal astrocytes respond with an endocannabinoid-induced endocannabinoid release (Hegyi et al., 2018). Thus, spinal glia can influence glial functions and synaptic neurotransmission at both excitatory and inhibitory synapses by releasing endocannabinoids (Stella, 2010; Katona and Freund, 2012; Ohno-Shosaku et al., 2012). Since endocannabinoids are among the most powerful antinociceptive mediators (Hohmann and Suplita, 2006; Woodhams et al., 2015; Finn et al., 2021), 2-AG released by SDH astrocytes and microglia can effectively target the pain processing neural networks in the SDH (Kato et al., 2012).

Participation of spinal astrocytes and microglia in the modulation of pain processing, however, can profoundly be influenced by their expression profiles. Reactive glia can release a set of inflammatory mediators including TNFα and IL-1β which can directly drive the development of central sensitization and a consequent persistent pain (Tang et al., 2021; Karavis et al., 2023). Our data confirmed that neuroinflammatory astrocytes and microglia in the SDH *in vivo* and *in vitro* increase the expression of COX-2 resulting in an increased capacity for the oxygenation of arachidonic acid derivatives including 2-AG. Indeed, spinal astrocyte-microglia co-culture increase the production of PGE2 in inflammatory environment. These results are in agreement with data obtained from rodent cortical astrocytes (Font-Nieves et al., 2012), although the substrate of COX-2 in that work has not been identified. When released in the SDH, prostaglandines directly drive the development of hyperalgesia by increasing the responses of nociceptive specific neurons to mechanical stimuli (Turnbach et al., 2002). PGE2 induces inflammatory hyperalgesia also by reducing synaptic inhibition of excitatory SDH neurons through an EP2 receptor-mediated pathway (Reinold et al., 2005). Thus, PGE2 alone released by reactive spinal glia in higher quantities may be sufficient to evoke inflammatory pain.

**Figure 9:**
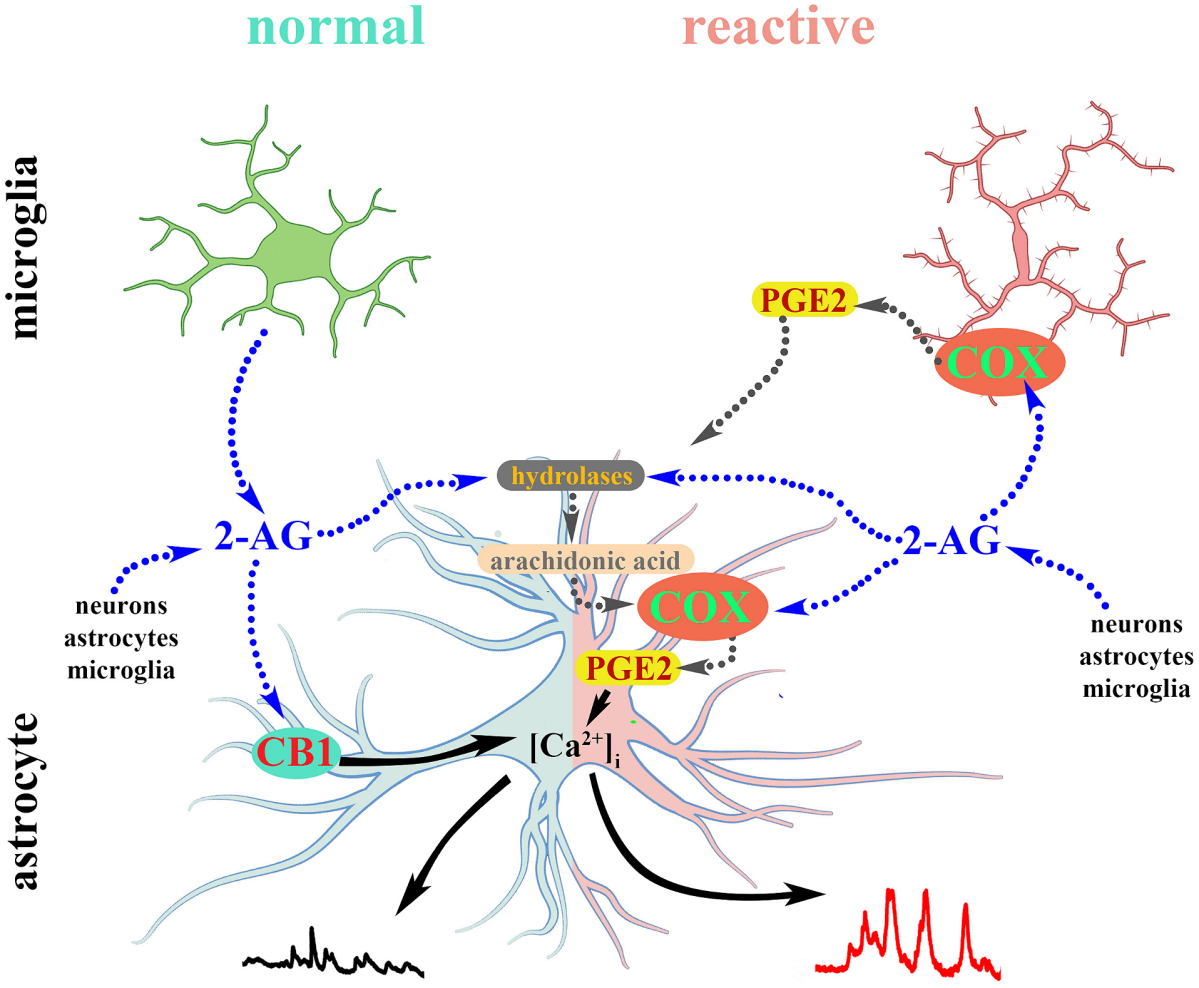
2-AG-induced calcium transients in normal astrocytes are mediated by CB1. Reactive glia convert 2-AG and its hydrolytic metabolite arachidonic acid into PGE2 which evokes aberrant calcium signals in reactive astrocytes.

An appropriate and timely release and elimination of transmitters influencing astroglial Ca^2+^ signaling is crucial to maintain a physiological environment in the central nervous system (Agulhon et al., 2008; Navarrete and Araque, 2011; Bazargani and Attwell, 2016). Disturbances of calcium signaling in astrocytes, accordingly, are directly associated with central nervous system disorders (Dong et al., 2018; Shigetomi et al., 2019; Bancroft and Srinivasan, 2021) including persistent pain (Ji et al., 2013; Cho and Huh, 2020; Tang et al., 2021; Xu et al., 2021). Our data suggests that COX-2 mediated oxidative metabolism of 2-AG is insignificant in resting astrocytes and microglia and barely influences astroglial calcium signaling. In contrast, neuroinflammatory spinal glia overexpress COX-2 and increase the conversion of 2-AG into PGE2. In our experiments, PGE2 proved to be 30% more efficacious and 55 times more potent than 2-AG at inducing calcium transients in astrocytes. Therefore, this alteration of lipid signaling seems to drive the appearance of aberrant calcium waves in reactive astrocytes which were abolished by the COX-2 inhibitor nimesulide. Based on the concentration-response curves for both ligands, we can speculate that, if released at a picomolar range, 2-AG has little effect on calcium signaling in astrocytes, but its conversion to PGE2 at the same concentration may result in robust calcium responses (Figure 9). Importantly, however, 2-AG content of the central nervous system varies from 4 to 12 nmol/wet tissue that equals 4–12 μM (Stella et al., 1997; Buczynski and Parsons, 2010) which can result in a production of significant quantities of PGE2.

PGE2 in concert with TNFα, ATP, interleukins and other gliotransmitters released by reactive glia then act as autocrine/paracrine mediators to drive the development of an inflammatory environment (Harada et al., 2015) and further activate not only an astrocyte-microglial network but also increase neuronal excitability which, in the SDH, results in central sensitization (Ji et al., 2018; Meesawatsom et al., 2020). In addition, PGE2 in our model is produced at the expense of 2-AG. Endocannabinoid release in SDH has been shown to decrease pain sensation (Rice et al., 2002; Sagar et al., 2010; Woodhams et al., 2015), therefore, a drop in the concentration of 2-AG can amplify the effects of PGE2 release at inducing central sensitization. Importantly, PGE2 is 34 times more soluble in water-based media than 2-AG (125 vs. 3.7 μM/L) suggesting that conversion of 2-AG to PGE2 extends the range of diffusion and increase the number of cells impacted by reactive glia. A broader area of diffusion, depending on the available prostaglandin receptors and elimination mechanisms, may result in diverse effects on different cells or cell types, and the clearance rate of PGE2 can also be manifold in the affected territory.

Intriguingly, an increased expression of COX-2 may influence the metabolism of another key endocannabinoid ligand, anandamide, due to the broad substrate specificity of the enzyme (Simard et al., 2022). Although we did not investigate if reactive spinal astrocytes convert anandamide into PGE2 ethanolamide, we can speculate that anandamide, similarly to 2-AG, undergoes a COX-2 dependent metabolic process in the presence of reactive glia. Anandamide inhibits nociception at the level of the spinal cord (Harris et al., 2000), therefore, a drop in anandamide concentration due to its cyclooxygenation may also lead to an increased nociceptive transmission. Importantly, both 2-AG and anandamide are hydrolyzed into arachidonic acid, which seems to be a double-edge sword in the modulation of Ca2+ signals. Arachidonic acid is a source of PGE2 production, therefore, it can effectively drive Ca2+ signals through a COX-2 dependent mechanism. On the other hand, arachidonic acid can also block Ca2+ oscillations by inhibiting store-operated Ca2+ entry (Sergeeva et al., 2003). Investigation of the effects of arachidonic acid on astroglial Ca2+ signals, however, was beyond the scope of our study.

Although our work focused on the role of spinal glia in inflammatory conditions, our data suggest that reactive astrocytes and microglia performing an endocannabinoid-prostanoid switch may play role not only in persistent pain but also in other neuroinflammatory disorders.

## Materials and methods

### Animals and preparation of tissue sections

Experiments were carried out on six, 12–15 weeks old male mice (NMRI, 30–35 g). All animal study protocols were approved by the Animal Care and Protection Committee at the University of Debrecen (license number: 12/2018 DEMAB), and were in accordance with European Community Council Directives. Animals were deeply anesthetized with sodium pentobarbital (50 mg/kg, i.p.) and transcardially perfused with physiological solution, followed by a fixative containing 2% paraformaldehyde (for fluorescent double immunostaining and for peroxidase-based immunohistochemistry) dissolved in 0.1 M phosphate buffer (PB, pH 7.4). After transcardial fixation, the lumbar segments (L4-L5) of the spinal cord were removed, post-fixed in their original fixative for 4 h, and immersed in 20% sucrose dissolved in 0.1 M PB until they sank. Spinal cords were sectioned at 40 μm on a cryostat, and extensively washed in PBS solution.

### Induction of peripheral inflammation

Peripheral inflammation was induced by the administration of Complete Freund’s Adjuvant (CFA, containing 1 mg of *Mycobacterium tuberculosis* (H37Ra, ATCC 25177), heat killed and dried, 0.85 mL paraffin oil and 0.15 mL mannide monooleate per mL; Hylden et al., 1989). CFA was dissolved in physiological solution in a ratio of 1:1 and injected into the right hind paw of three mice. Based on previous studies, inflammation and mechanical hypersensitivity develops 4 days after CFA injection (Gajtkó et al., 2020). Therefore, animals were sacrificed on day 4 following the administration of CFA.

### Antibodies

For single and double immunostaining, the following primary antibodies were used: mouse-anti-GFAP (1:2000, Synaptic Systems, Cat. No: 173011), guinea pig-anti-Iba1 (1:500, Synaptic Systems, Cat. No: 234013), goat-anti-DGL-α (1:200, Frontier Institute, Cat. No: MSFR101310), guinea pig anti-CB1 (diluted 1:200; Frontier Institute, Cat. No: CB1-GP-Af530-1) or rabbit-anti-COX-2 (1:200, Synaptic Systems, Cat. No: 12282). The specificity of primary antibodies has been extensively tested in earlier studies (Hegyi et al., 2009, 2012; Chopra et al., 2019).

### Single immunostaining

The distribution of immunoreactivity for GFAP, DGL-α, CB1 and COX-2 in the SDH of naïve and CFA-treated adult NMRI mice was studied performing a single immunostaining protocol. Before applying antibodies, free-floating sections were first treated with 50% ethanol for 30 min followed by 10% normal goat or rabbit serum (Vector Labs) for 50 min. Sections were then incubated in mouse-anti-GFAP, guinea pig-anti-Iba1, goat-anti-DGL-α, guinea pig anti-CB1 or rabbit-anti-COX-2 for 48 h at 4°C, followed by a biotinylated goat-anti-rabbit, goat-anti-mouse, goat-anti-guinea pig or rabbit-anti-goat IgG (1:200; Vector Labs, Burlingame, CA, United States) for 4 h at 4°C. The sections were then transferred to an avidin biotinylated horseradish peroxidase complex (1:100, Vector Labs) for 1 h at room temperature. The immunoreaction was visualized with a 3,3′-diaminobenzidine (Sigma, St Louis, MO, United States) chromogen reaction. Antibodies were diluted in 10 mM Tris-phosphate-buffered saline (TPBS, pH 7.4) supplemented with 1% normal goat serum (Vector Labs). Sections were mounted on glass slides and covered with a neutral medium after dehydration.

### Isolation of spinal astrocytes and microglia

Primary spinal astrocytes and microglia were cultured from 4 days old NMRI mice. Following a decapitation of animals and the removal of spinal cord, samples were transferred into ice-cold dissecting buffer (136 mM NaCl, 5.2 mM KCl, 0.64 mM Na2HPO4, 0.22 mM KH2PO4, 16.6 mM glucose, 22 mM sucrose, 10 mM HEPES supplemented with 0.06 U/mL penicillin and 0.06 U/mL streptomycin). After the removal of meninges, spinal cords were first incubated in a solution containing 0.025 g/mL bovine trypsin (catalog no.: T4799, Sigma, St Louis, United States) for 30 min at 37°C, followed by a Dulbecco’s Modified Eagle’s Medium containing high glucose (catalog no.: D6429, Sigma, St. Louis, United States) and 10% fetal bovine serum (catalog no.: F2442, Sigma, St. Louis, United States) to stop the action of trypsin. Samples were suspended, and the cell suspension was filtered on a nylon mesh. Thereafter, the isolated cells were transferred into 24-well plates coated with 0.3 mg/mL poly-L-lysine. For all experiments, cell density was 6 × 10^5^/ml. Cells were maintained for 7–12 days at 37°C in a CO2 incubator (CO2 concentration: 5%, humidity: 95%), and the medium was changed every second day. Contaminating neurons were identified by immunocytochemical staining against a marker specific for neurons (NeuN), whereas astrocytes and microglia were identified by GFAP and Iba1 immunoreactivity, respectively. Cultures with a purity of at least 80% (that is, the proportion of GFAP and Iba1 positive cells was more than 80%) were used for the experiments.

### Induction of reactive glia

Astrocytes and microglia respond to LPS treatment with a profound change in their protein expression profile resulting in the formation of reactive astrocytes. Importantly, rodent astrocytes lack receptors and downstream signaling components required for LPS activation (TLR4 and MYD88), indicating that LPS results in the formation of reactive astrocytes through microglial activation (Liddelow et al., 2017). Therefore, 10–12 day old cell cultures were treated with 50 ng/mL LPS for 2 h (30 min, 1 and 2 h in Simple Western experiments) to induce reactive glia. Cells were kept in Dulbecco’s Modified Eagle’s Medium containing high glucose (catalog no.: D6429, Sigma, St. Louis, United States), enriched in 10% fetal bovine serum (catalog no.: F2442, Sigma, St. Louis, United States) in a CO2 incubator for the time of LPS stimulus.

### Fluorescent immunocytochemistry

Tissue sections obtained from naïve and CFA-treated adult NMRI mice, as well as control and LPS-stimulated cell cultures were incubated in a mixture of mouse-anti-GFAP or guinea pig-anti-Iba1 and rabbit-anti-COX-2. The sections and cultures were then treated with goat anti-rabbit IgG conjugated with Alexa Fluor 488 (1:1000, catalog no.: A11034, Invitrogen) and goat anti-mouse IgG conjugated with Alexa Fluor 555 (1:1000, catalog no.: A21422, Invitrogen) secondary antibodies. Prior to the antibody treatments, the samples were incubated in 10% normal goat serum (Vector Labs) for 50 min. For the dilution of antibodies, PBS (pH 7.4) containing 1% normal goat serum (Vector Labs) was used. Sections were mounted on glass slides. Cultures and sections were covered with VectaShield-DAPI (Vector Labs).

### Imaging and image analysis of single immunostained sections

Image aquisition of single immunostained sections was carried out with an Olympus DP72 camera built on an Olympus BX53 microscope using a 20x dry objective lens (NA: 0.40). Grayscale intensity histogram profiles were taken from 3 randomly selected immunostained spinal dorsal horns of naïve and CFA-treated animals. Pixel intensities in laminae I-II, defined as a 100-μm-thick band of spinal dorsal horn from the border between white matter and gray matter, were measured in Fiji. Illustrations were prepared in Originpro 9 and Adobe Photoshop CS5.

### Imaging and image analysis of double immunostained specimen

Immunostained sections and cultures were investigated using an Olympus FV3000 confocal microscope with a 10x dry objective (NA: 0.25) or a 60x oil-immersion lens (NA: 1.4). Laser power, confocal aperture and gain were identical for all the samples to be compared. Single 1-μm-thick confocal optical sections or short image stacks with an overlap of 0.5 μm were recorded and further analyzed in Imaris and Fiji. Illustrations were prepared using Adobe Photoshop CS5 software.

The colocalization between immunostainings for COX-2 and GFAP or Iba1 was analyzed in double stained spinal cord sections in Fiji. Quantitative measurement was carried out in three randomly selected spinal dorsal horns of naïve and CFA-treated animals, ipsilateral to the treatment. Laminae I-II of spinal cord were defined as the most superficial 100-μm-thick band of the dorsal grey matter. Colocalization of immunostainings was identified in Fiji based on the overlapping pixels between the corresponding channels.

The colocalization of COX-2 and GFAP or Iba1 was analyzed in cell cultures in Imaris. The analysis of astrocyte cultures was performed on three independent cultures. All GFAP or Iba1 positive cells in five randomly selected fields of views from each culture were investigated for co-localization with COX-2-positive particles.

### Capillary electrophoresis immunoassay (simple Western)

Samples from unstimulated and LPS-treated cell cultures were collected and lysed in a radioimmunoprecipitation (RIPA) lysis buffer containing Pierce protease inhibitor tablet (catalog no.: A32963, Thermo Fisher) and 1% NP-40. Prior to the collection of samples, cells were washed 2x in a medium lacking fetal bovine serum. The protein content of samples was measured using Pierce BCA protein assay kit (catalog no.: 23225, Thermo Fisher). All samples were denatured in the presence of 1X Fluorescent Master Mix (PS-FL01-8, ProteinSimple) for 5 min at 95°C based on the manufacturer’s instructions. Total protein fractions (1 μg/mL) were assayed using a ProteinSimple WES automated capillary-based electrophoresis instrument. A WES Separation Module protocol (ProteinSimple) was performed using a 12–230 kDa separation module (SM-W004, ProteinSimple) and an anti-rabbit detection module (DM-001, ProteinSimple). The COX-2 protein was detected using rabbit anti-COX2 (1:25, Synaptic Systems) primary antibody. Results were analyzed and the area under the curve (AUC) was calculated in Compass for SW software (v6.1.0).

### Measurement of PGE2 concentration

PGE2 concentration in control and LPS-treated cell cultures was measured using a commercially available Prostaglandin E Metabolite ELISA competitive assay (Cayman Chemical, catalog no.: 514531), which converts prostaglandines to a single, stable derivative. Before performing the assay, samples were stored at −80°*C. prior* to the experiments, proteins were removed from the samples using an acetone precipitation. Thereafter, acetone was removed and supernatant was dried using a nitrogen evaporator device. Samples were then resuspended in ELISA buffer and assays were performed according to manufacturer’s recommendation. Results were evaluated in Cayman’s ELISA analysis tool.^1^ The concentrations of samples were calculated using a linear regression fit.

### Fluorescent Ca^2+^ imaging

Before calcium imaging experiments, astrocyte-microglia co-cultures were loaded with 1 μM Fluo-8-AM in the presence of 0.01% pluronic 127 at room temperature for 30 min. Ca^2+^ imaging was performed using a differential spinning disk (DSD2, Andor Technology) connected to an Andor Zyla 5.5 sCMOS camera. The imaging setup was built on an Olympus IX-81 inverse microscope. Using a 20× objective (NA: 0.40), images of 540 μm × 306 μm field of view, which contained around 15 to 40 cells were acquired at 10 frames per second in Andor iQ3 software. Astrocytes were distinguished from microglia based on their morphology, verified by a GFAP immunostaing following Ca2+ imaging experiments. Traces from cells negative for GFAP were discarded. Fluo-8-AM loaded astrocytes were excited at 488 nm and emission was detected at 520 nm. Acquisition parameters (illumination intensity, exposure time, readout time, frame rate) were identical for all measurements. Changes in fluorescence intensities were measured for 10 min over visually identified processes of astrocytes by drawing freehand ROIs around them. Changes in the intracellular calcium concentration were estimated as changes of the fluorescence signal over baseline (ΔF/F0, where F0 was the average initial fluorescence). Changes in fluorescence intensities of an astroglial process was considered a calcium signal (either spontaneous or treatment-induced) if ΔF/F0 was three times the standard deviation of the baseline for at least five consecutive images. Recorded data were analyzed in Microsoft Excel 365 (Microsoft). FFT filtering to reduce noise and calculation of area under the curve (AUC) were performed in Originpro 9 (Originlab, Northampton, MA, United States).

### Data analysis

Statistical analysis was performed using Originpro 9 software. Data for Figure 1 were analyzed using the Kolmogorov–Smirnov test. Analyses of data for Figures 2, 4 were performed using the two-tailed non-parametric Mann–Whitney U test. Data for Figures 3, 5, 7 were analyzed using the two-way Anova with a Bonferroni correction. Cluster analysis for Figure 8 was performed using K-means cluster analysis. Differences were considered significant when the p level was <0.05. Data are presented as mean ± standard error of the mean (SEM) Differences were indicated in the figures as follows: * if *p* < 0.05; ** if *p* < 0.005; *** if *p* < 0.001.

## Data availability statement

The original contributions presented in the study are included in the article/Supplementary material, further inquiries can be directed to the corresponding author.

## Ethics statement

The animal study was approved by Animal Care and Protection Committee at the University of Debrecen. The study was conducted in accordance with the local legislation and institutional requirements.

## Author contributions

KD: Data curation, Formal analysis, Investigation, Methodology, Project administration, Writing – review & editing. AB: Data curation, Investigation, Methodology, Writing – review & editing. IP: Formal analysis, Investigation, Writing – review & editing. PS: Conceptualization, Funding acquisition, Methodology, Writing – review & editing. ZH: Conceptualization, Formal analysis, Investigation, Methodology, Project administration, Writing – original draft, Writing – review & editing, Funding acquisition.

## Footnotes

^1^ https://www.caymanchem.com/analysisTools/elisa
