# Supplementary figures and images for: Reactive spinal glia convert 2-AG to prostaglandins to drive aberrant astroglial calcium signaling
Source: Front Cell Neurosci. 2024 May 9;18:1382465. doi: 10.3389/fncel.2024.1382465 (PMC11112260; doi:10.3389/fncel.2024.1382465)

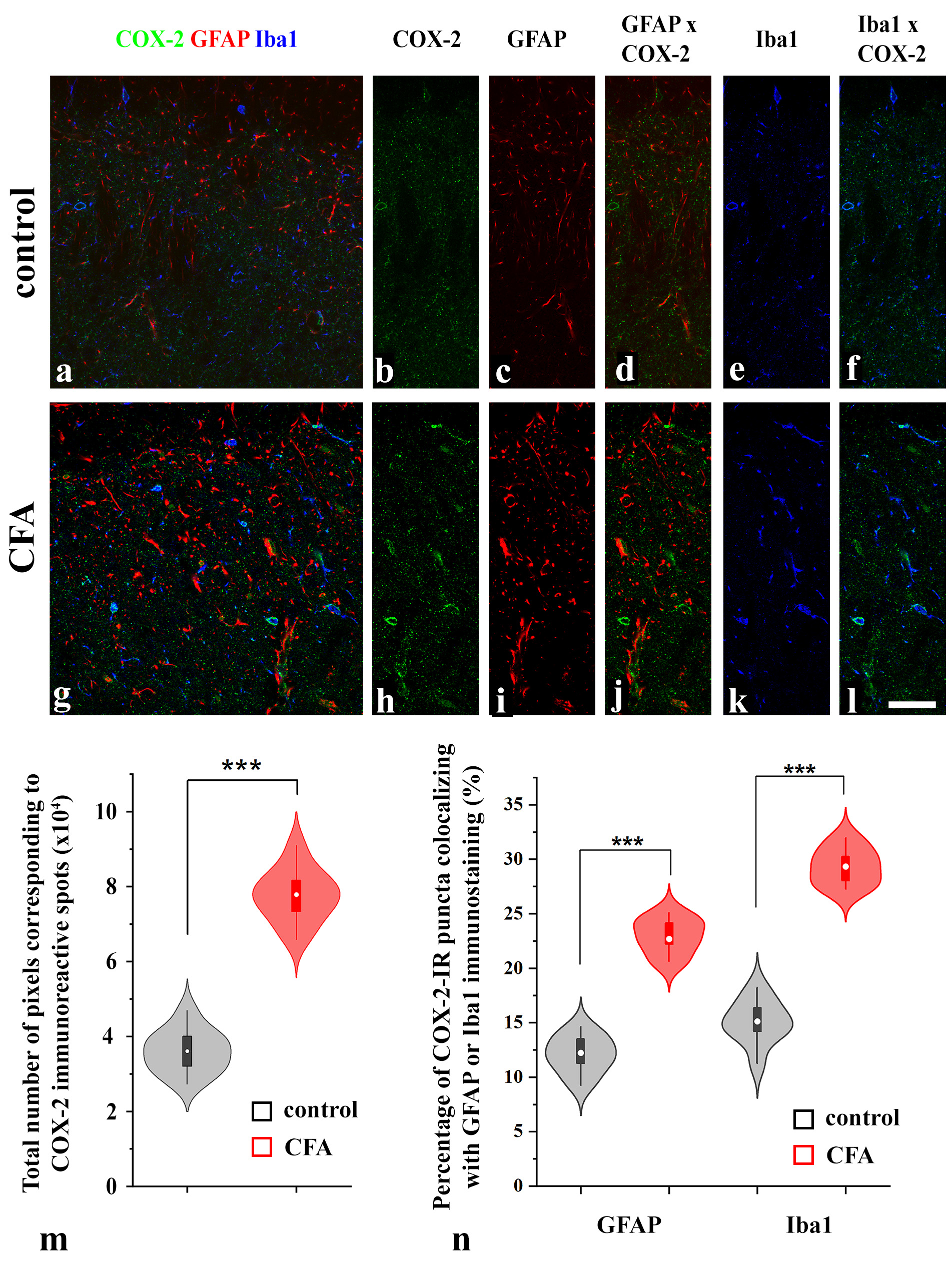

Supplement: Supplementary file 1 [file Image_1.JPEG]
